# Computer-Assisted Update of a Consumer Health Vocabulary Through Mining of Social Network Data

**DOI:** 10.2196/jmir.1636

**Published:** 2011-05-17

**Authors:** Kristina M Doing-Harris, Qing Zeng-Treitler

**Affiliations:** ^1^University of UtahDepartment of Biomedical InformaticsSalt Lake City, UTUnited States

**Keywords:** Consumer health information, vocabulary, natural language processing, automatic term recognition, data mining, social networking

## Abstract

**Background:**

Consumer health vocabularies (CHVs) have been developed to aid consumer health informatics applications. This purpose is best served if the vocabulary evolves with consumers’ language.

**Objective:**

Our objective was to create a computer assisted update (CAU) system that works with live corpora to identify new candidate terms for inclusion in the open access and collaborative (OAC) CHV.

**Methods:**

The CAU system consisted of three main parts: a Web crawler and an HTML parser, a candidate term filter that utilizes natural language processing tools including term recognition methods, and a human review interface. In evaluation, the CAU system was applied to the health-related social network website PatientsLikeMe.com. The system’s utility was assessed by comparing the candidate term list it generated to a list of valid terms hand extracted from the text of the crawled webpages.

**Results:**

The CAU system identified 88,994 unique terms 1- to 7-grams (“n-grams” are n consecutive words within a sentence) in 300 crawled PatientsLikeMe.com webpages. The manual review of the crawled webpages identified 651 valid terms not yet included in the OAC CHV or the Unified Medical Language System (UMLS) Metathesaurus, a collection of vocabularies amalgamated to form an ontology of medical terms, (ie, 1 valid term per 136.7 candidate n-grams). The term filter selected 774 candidate terms, of which 237 were valid terms, that is, 1 valid term among every 3 or 4 candidates reviewed.

**Conclusion:**

The CAU system is effective for generating a list of candidate terms for human review during CHV development.

## Introduction

Controlled vocabularies play an important role in the development of biomedical informatics applications because data used by clinical, bibliometric, and research applications need to be coded for easy retrieval and analysis. Research and development activities have been carried out to provide standardized health vocabularies, for example, SNOMED (Systematized Nomenclature of Medicine) and LOINC (Logical Observation Identifiers Names and Codes). In the past, these vocabularies focused on the language of professionals, but lately consumer health vocabulary (CHV) [1] has been rising in prominence, and, consequently, CHV research has gained recognition.

Controlled vocabularies require maintenance and updating due to the continuing evolution of language itself [2-4]. This evolution has been seen for centuries in the regular update and revision of dictionaries [5,6]. Controlled vocabularies serving electronic applications are no exception. The demand for maintenance and updating of vocabularies is particularly high in areas related to ongoing research and development. As new findings emerge, new words are added to the vocabulary. In health care especially, there is a constant stream of new names (eg, new medications, disorders, and tests) [7].

Frequently, new health terms used by professionals migrate, in some form, into popular parlance. For example, the term *mass spectrometer* was unheard of 30 years ago, but a number of lay people now could identify it as a piece of lab equipment. Although *deoxyribonucleic acid* may be confusing, DNA is in the vocabulary of school-aged children. The media also plays a role in term migration. For example, in 2009, media coverage introduced new vocabulary words such as *pandemic*, *swine flu*, *H1N1*, *energy expenditure*, and *single-payer system* into popular speech. Similarly, the meaning and popularity of health terms change or evolve in the lay use. For example, it is common for lay people to use the term *anorexia* to refer to the concept *anorexia nervosa*, though in medical literature *anorexia* refers only to the loss of appetite.

To be effective, a CHV must keep pace with changes in the language used by consumers [1]. This paper describes a computer-assisted update (CAU) system that uses an online social network as a living corpus of health-related terms. The system parses and screens terms using the natural language processing (NLP) techniques of dictionary lookup and automatic term recognition. New candidate terms are thereby identified for inclusion in the open access and collaborative (OAC) CHV.

### Background

In this background section, we will first briefly review the prior research and current practice for updating controlled health vocabularies. Next, we will discuss the automated methods used to identify valid terms from text corpora. Then, we will switch focus to provide background information on the OAC CHV research. Finally, we will describe the rationale behind using a live corpus with automated term identification for updating the OAC CHV.

### Updating Controlled Health Vocabularies

Prior research has found that nearly all large controlled health vocabularies have similar core maintenance procedures [8]. Bakhshi-Raiez et al describe a framework for the maintenance of controlled health vocabularies. They refer to controlled health vocabularies as medical terminology systems (TSs). Their framework consists of four components. The primary component of their framework is “execution.” This covers the core activities of the maintenance process including: collection of proposals for changes, validation of the proposals for changes, implementation of changes, verification of changes, documentation of proposals and implemented changes, and version management. The three other components, namely “process management,” “change specification,” and “editing tools” act in support of “execution.” Bakhshi-Raiez et al conducted a survey of 37 TSs. They divided the group of TSs into quartiles based on the number of concepts included in each system. The quartile relevant to this paper is quartile IV, which included systems with more than 46,155 concepts. Quartile IV would include the OAC CHV, which has 58,319 concepts. For the execution component, almost all of the quartile IV systems satisfied the main criteria, that is, 67% included standardized change proposals, 100% validated the change proposals, 100% had maintenance teams that verified accepted proposals, 100% had structured and standardized documentation, 100% documented changes made, and 100% produced new versions with unique id’s, while only 70% produced twice yearly updates. The CAU system we describe here is designed to automate the production and collection of change proposals and then assist with the validation of those proposals.

The current practice for the generation and collection of proposals for TS changes and their validation typically involves collecting proposals via email or Internet and having a team of specialists validate them. For example, there is a Web-based Semantic MediaWiki system for maintaining entries in the National Cancer Institute Metathesaurus [9]. The SPECIALIST Lexicon included in the Unified Medical Language System (UMLS) collects words from literature as well as multiple dictionaries [10]. The medical subject headings (MeSH) section staff continually revises and updates the MeSH vocabulary based on scientific literature in emerging areas of research, defines these terms within the context of the existing vocabulary, and recommends their addition to MeSH [11]. In a personal communication, Stuart Nelson, the head of MeSH, estimated that 20% of his time is devoted to updating and revision. There are also six full time MeSH analysts. Clearly, vocabulary maintenance is a labor-intensive process, one whose efficiency could be improved by the proposed CAU system. The first step to automation would be the generation and collection of proposals for changes, a step that lacks standardization in one-third of all large vocabularies’ maintenance procedures [8].

### Automatic Term Recognition in the Biomedical Domain

One method of automatically generating change proposals is to identify valid candidate terms in a text corpus through automatic term recognition (ATR) [12,13]. ATR studies overlap with the discipline known as named entity recognition (NER). ATR refers to systems that search for general types of terms as opposed to named entities. A term becomes a named entity when it is mapped to an ontology or dictionary of terms, which gives the term meaning in a context outside of the document in which it is found. General terms have no such wider meaning. Examples of biomedical NER systems include Termoid, MetaMap and Bio-tagger [14-16]. Examples of biomedical ATRs are Collier et al’s hidden Markov model for identifying gene names and gene products, as well as Frantzi et al’s “C-value” and Zeng et al’s “termhood” score [17-19].

Since C-value and termhood scores are used in our study, we will briefly describe them here. The C-value equation uses part of speech-tagged data and restricts candidate terms to noun phrases. The best results are obtained with an open linguistic filter that returns noun phrases, which include multiple adjectives and nouns [18]. C-value is then calculated using the frequency of occurrence of the candidate term combined with its frequency of occurrence as part of other, longer candidate terms, along with the number of longer candidate terms and their lengths. Expanding upon the C-value, the termhood logistic regression equation (termhood score) was developed by Zeng et al to identify multi-word consumer health terms including those that are not noun phrases [19]. The features used to train the logistic-based model include parts of speech of term components, frequency of occurrence of candidate terms, as well as frequency of occurrence of said candidate terms in both larger and smaller alternative candidate terms. Zeng et al compared C-value and termhood score ratings using strings that had already been human reviewed and found that termhood score outperformed C-value on their dataset [19].

### Consumer Health Vocabulary

The development of this CAU system is part of the OAC CHV research program. The OAC CHV was developed using a phased, distributed, user source-based approach [1]. To incorporate new terms, seven human review criteria were established [19]: (1) CHV terms should be syntactic constituents or phrases such as noun phrases or adjectival phrases; (2) CHV terms should have independent semantics and should not only occur as a part of longer valid terms or as a part of wild card searches; (3) CHV terms should be specific to the medical domain; (4) CHV terms should function as semantic components; (5) “n-grams” (n-grams are n consecutive words within a sentence) representing UMLS concepts are considered to be CHV terms, but CHV terms may represent non-UMLS concepts; (6) CHV terms may be eponymous forms; and (7) CHV terms may include spelling errors. These criteria guide the human review in this study; the current version of the CHV contains 152,778 entries, representing 58,319 concepts.

### Live Corpora

From the beginning, research on CHV relied on text corpora containing consumer utterances. Although most of the text corpora were collected from live sources such as patient email, online forums, query logs, and social networks [16-20], they were treated as static datasets for analysis. In this study, we aimed to directly tap into the live sources. Due to the extremely fast growth of social network sites, including health-related social network sites and their public availability [21], we chose to test the CAU system on the social networking site PatientsLikeMe. Our lab has a collaborative relationship with PatientsLikeMe, which facilitated permission to use the site. The CAU system, however, could also work with other types of live sources.

PatientsLikeMe.com is an online community built to support information exchange between patients. The site provides customized disease-specific outcome and visualization tools to help patients understand and share information about their condition [22,23]. The private pages of the site are designed for patients to enter symptoms and track their disease. The public pages, on the other hand, include information provided by the site management and excerpts of information shared by users. The public pages thus contain language used by professionals as well as lay people. An example of language used by professionals would be, “ALS, or amyotrophic lateral sclerosis, is a neurodegenerative disease caused by the degeneration of motor neurons.” An example of language used by a lay person would be, “…The first thing that I thought might be your problem is malnutrition. Man, you’re losing weight crazy fast. I think you better consider getting peg tube if you desire.” By sticking to the public pages, we plan on tapping into this social networking aspect of the site without breaching the privacy of the users. 

## Methods

We devised the CAU system to mine Web content using a combination of NLP methods: dictionary-lookup, C-value ATR, and termhood ATR. The goal was to discover new health-related terms used by consumers but not yet included in our existing vocabulary. The best candidate term list should contain a small number of terms while providing a reasonably high yield of valid terms after the human review.

### System Architecture

**Figure 1:**
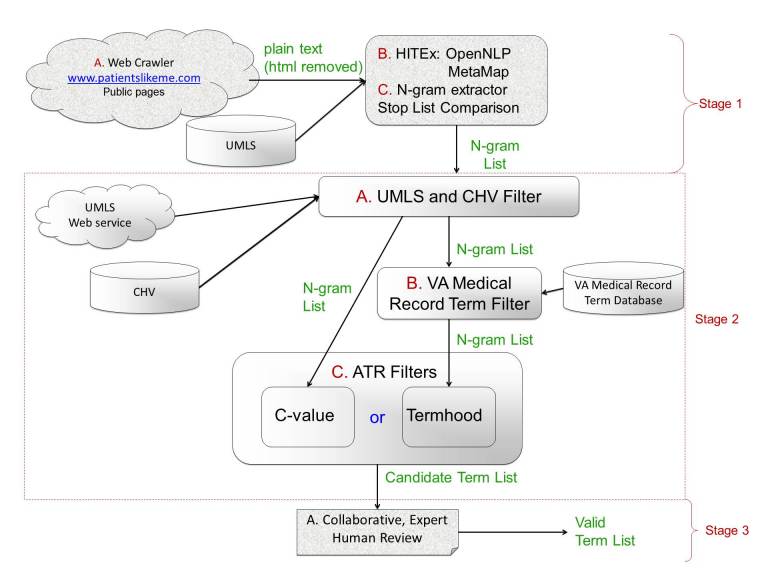
CAU System Diagram

The CAU system architecture is shown in Figure 1. It consists of three processing stages, stage 1, stage 2, and stage 3. Stage 1 is the stage in which raw text is obtained, parsed, and n-grams, that is, groups of words, are extracted. This stage involves three substages: crawling, parsing, and n-gram extraction.

#### Crawling

In the crawling substage, the system crawls public pages on the Web. Crawling consists of navigating to the home page, collecting all the links to other pages in a queue, navigating to those pages in turn, and adding any links found to the end of the queue. This loop continues until the end of the queue or until a predefined number of pages has been visited. The remaining content of each page is processed by removing HTML tags, adding periods to the ends of text blocks followed by more than one new line, and saving the resultant text.

#### Parsing

In the parsing substage, the system uses the open-source natural language processing application Health Information Text Extractor (HITEx) [24] to identify parts of speech, noun phrases, and named entities. HITEx is an NLP system, which contains an OpenNLP parser and uses MetaMap for NER.

#### N-gram Extraction 

In the n-gram extraction substage, the system extracts n-grams (1- to 7-grams) with overlap. Overlap means a word can be included in more than one n-gram. The n-grams are filtered to retain n-grams identified by HITEx as noun phrases, n-grams, which contain a verb (ie, potential verb phrases), and n-grams that contain the word *symptom*. N-grams that include numbers or symbols are excluded at this point. This linguistic filtering strategy is based on Frantzi et al’s finding that the C-value ATR produced better results with an open linguistic filter [18]. A stop list (ie, a list of terms that should be ignored) was created from a list of the 1000 most common phrases in English (eg, *a little*, *a few*, *we like it very much*) [25]. Terms found on the stop list are excluded and frequency information is gathered at this point.

Stage 2 is the stage in which further NLP techniques are used to identify candidate terms on the n-grams list. This stage consists of three substages, two dictionary-type look up stages, the UMLS/CHV filter substage and the VA medical record term filter substage, and one ATR stage, the ATR filter substage.

##### UMLS/CHV Filter

Given our interest in discovering new terms, the n-grams are looked up in the current CHV list and the UMLS Metathesaurus. To insure the most up to date version of UMLS (2010AA) was used, n-grams were checked using the UMLS Web service. Those n-grams that were not present in UMLS or CHV were denoted non-CHV.

##### VA Medical Record Term Filter

To filter the nonmedical terms from the non-CHV n-grams, we looked them up in a database of 70,000 medical records of patients obtained from the US Department of Veteran’s Affairs of patients with amyotrophic lateral sclerosis (ALS), Parkinson’s, and multiple sclerosis (MS) dated from January 1, 1998, through December 31, 2008. These records contain a broad spectrum of medical topics and note types. They are not limited to neurology or the three diseases. These records were obtained by another group in our department with internal review board (IRB) approval. IRB approval was given for a member of that group to compare terms to this database for us, returning a yes/no answer. All terms, which returned *yes* were entered into a database for future comparisons. We will refer to this database as the VA medical record term database.

##### ATR Filters

Calculated are two ATR scores, that is, termhood and C-value. The termhood score was calculated using the logistic regression equation described in Zeng et al [19]. The C-value is calculated using the equation described in Frantzi et al [18]. 

The third stage is the human (expert) review stage. In this stage, candidate terms are submitted for collaborative expert review. To aid this process, we created an interactive website for the OAC CHV. Approved reviewers can access the site and recommend URLs for inclusion in the crawl, review candidate terms, review recent candidate term comments, and review CHV preferred names. While reviewing candidate terms, the reviewer can vote for or against a term’s inclusion in the OAC CHV, see all terms’ frequencies and votes, and get three examples of a term in the context of the webpage on which it was found. Reviewers can also comment on a candidate term without registering a vote. Each reviewer can vote for a term only once. The public may comment on the candidate terms on the CHV Wiki by browsing the term list, choosing candidate terms, and clicking the term on which they would like to comment.

### Evaluation

The system was evaluated by crawling the PatientsLikeMe.com website, examining the candidate terms identified and calculating valid term yield (ie, percentage of total candidate terms which are valid terms). For the purposes of this paper, the final stage of collaborative human review was replaced by the creation of a valid term list, which functions as the gold standard for this study. The valid term list was manually extracted from the webpages by the first author and filtered to exclude terms already represented in the UMLS/CHV. Reviewing the first 300 pages encountered in the crawl produced a valid term list containing 651 terms, which we considered sufficiently large for the purposes of this experiment. Therefore, we restricted the processing by the system to those pages.

To assess the accuracy of the valid term list, a panel of expert reviewers (two physicians and two allied medical personnel) reviewed 100 random non-CHV terms found in our VA medical record term database from the initial parse of the webpages. Each expert’s agreement with the gold standard valid term list was assessed using the balanced F-measure discussed in Hripcsak and Rothschild [26]. The F-values found were 0.94, 0.86, 0.94, and 0.91, indicating that terms chosen as valid were indeed valid and that valid terms were not being missed.

## Results

### Stage 1

The crawler visited the PatientsLikeMe.com website (marked A in Figure 1) public pages only. The parsing and n-gram extraction phases (marked B and C in Figure 1) found 88,994 n-grams. The n-gram list contained all 651 terms from the valid term list.

### Stage 2

In the UMLS/CHV filter phase 1045 (1% of the total) n-grams were found in the CHV/UMLS. The total number of non-CHV terms remained large at 87,949.

The VA medical record term filter phase filtered out most of the n-grams. It eliminated all but 923 n-grams (99% reduction) and all but 215 terms from the valid term list (67% reduction).

The eliminated valid term list terms in this phase were, for the most part, long (eg, *sub mandibular injection paralyzed swallow muscles*), brand names (eg, *Nurofen*), combination terms (eg, *lipodystrophy lipoatrophy*), or biochemical terms (eg, *L-methyfolate Metafolin*). The loss of these terms was concerning, but it is possible that they would be found if a comparison was made with a larger database of medical records. Other valid term list terms excluded were consumer terms (eg, *brain fog* and *loss of time*), which may not typically be recorded in medical records. 

**Figure 2:**
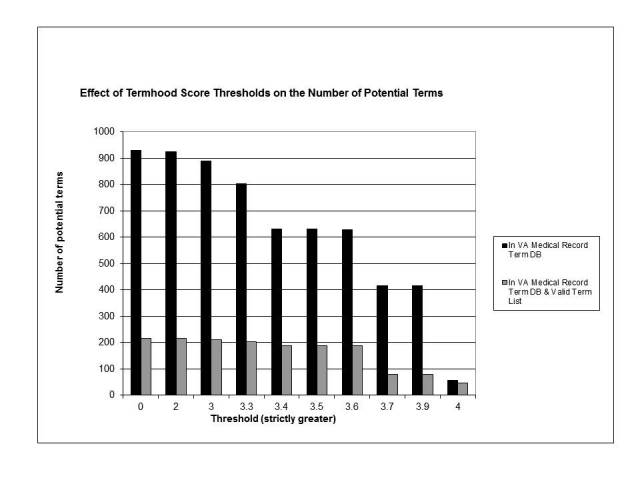
Effect of termhood score thresholds on the number of candidate terms

**Figure 3:**
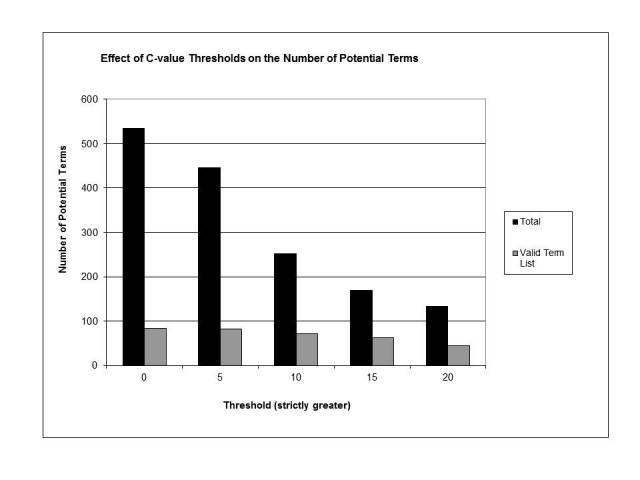
Effect of C-value score thresholds on the number of candidate terms

In the ATR filter phase, two filters were built by applying a threshold to the ATR scores. The first filter used was based on termhood score and was applied to each non-CH n-gram found in the VA medical record term database. From Figure 2 it can be seen that the number of terms above the threshold dropped gradually. The best yield of valid terms was achieved with thresholds between 3.4 and 3.6. Choosing the higher of these thresholds (3.6) identified 622 candidate terms (99.3% cumulative reduction) of which 189 were from the valid term list (69% cumulative reduction), a 30% valid term yield. The excluded valid terms include terms such as *asthenia symptom* and *breast-feeding*.

The second filter was based on C-value and was applied to all non-CHV n-grams. From Figure 3 it can be seen that the number of terms above this threshold also dropped gradually. The best yield of valid terms was found at a threshold of 15 (170 terms, 93.7% reduction, with 62 from the valid term list, 90% reduction), a 36% yield. This yield was higher than the termhood with VA medical record term filter, but the number of candidate terms returned was much lower. Some of the valid terms excluded here were ill-formed phrases that included the word *symptom* (eg, *asthenia symptom*, *cramps symptom*), while others were potentially more concerning (eg, *staggering walk, inability to raise the foot*).

When the C-value threshold was combined with the termhood threshold the number of candidate terms increased while keeping the valid term yield around 30%, the number of terms remaining was 774 with 237 valid terms. Combining the filters caught 48 more valid terms over the termhood filter alone, including *cold legs below knee, augmentative speech device, and acid reflux GER gastroesophageal reflu*x.

The results are summarized in Table 1. Table 2 contains a sample list of candidate terms identified.

**Table 1:** Results at each stage of CAU processing

Processing Phase	System Stage (as labeled in Figure 1)	Candidate Terms	Valid Terms
1. Initial parse	1	88,994	651
2. CHV/UMLS filter	2A	87,949	651
3. VA medical record term filter	2B	923	215
4. ATR filter 1: termhood score (threshold 3.6) (VA medical record terms only)	2C	622	189
5. ATR filter 2: C-value (threshold 15) (all terms)	2C	170	62
Phase 4 and 5 filters combined	2C	774	237

**Table 2:** Sample list of valid and invalid candidate terms identified (displayed in no particular order and in the format in which they were found)

Sample of Candidate Terms Identified		
Valid Terms	Invalid Terms	
manage Side effects Bi-Pap Weakness in Hands Devic’s Neuromyelitis Optica	Does not protect PRO survey Motorized recliner PRO 0 MSA Multiple System fitness depending is particularly Seizures grand mal saliva control	acidify Permobil Levetiracetam Treatment Report Pilates Treatment Report Equate Acetaminophen all the time Hoveround cope Seage III GR

Stage 3, the expert human review stage, was preempted by the use of the valid term list as gold standard in this study.

## Discussion

### Principal Result

We developed a vocabulary maintenance system and tested it on the PatientsLikeMe.com website. The system first identified a very high number of n-grams (n = 88,994) and then created a candidate term list of a reasonable size (n = 774) with a relatively high valid term yield (31% or 237/774). The system and the experiment are a proof-of-concept for procuring new terms using living corpora and ATR to aid vocabulary maintenance. 

The system utilized NLP methods including parsing, dictionary look up, comparison with a medical record database, and ATR to filter out both the n-grams that were not related to health and ill-formed sentence fragments. Following all the filtering, the reviewers found 1 valid term among every 3 or 4 candidate terms reviewed. This is considerably better than the initial n-gram list which would have returned on average 1 valid term for every 137 candidate terms.

The system will become more efficient after each maintenance cycle. All candidate terms rejected for inclusion will be added to the stop list, which should decrease the number of candidate terms. For instance, following the experiment described in the paper we conducted a second crawl of 300 pages and obtained 240 candidate terms for human review with 71 potentially valid terms, which maintained a yield of 30% (71/240). However, since 88 of the candidate terms were place names expanding the stop list to include place names would reduce the candidate term list to 212 with 71 potentially valid terms, a valid term yield of 33% (72/212).

There have been previously reported higher yields using C-value and termhood scores [18,19]. The yield, however, is sensitive to the data and task involved in each study. Spasic et al used C-value to extract terms from full-text journal articles with a reported yield of 61%. However, they targeted all valid terms instead of new terms (ie, terms not yet included in a vocabulary), which are fewer and harder to find. In our own previous study to identify new CHV terms, both termhood score and C-value score were used. The termhood score yield was 38% and the C-value score was even lower. The data set used in that study was the query log to MEDLINEPlus. Compared with query logs, PatientsLikeMe pages contain more “noise” (ie, terms that are similar in structure to those we seek but are not health-related), which increases the number of candidate terms found. We chose not to use only either C-value or termhood scores alone on these data because the results produced were much lower than the 31% we report here.

### Implications for the System

The results of this study point to the necessity of using both the termhood and C-value methods. The termhood score required first matching terms with the VA medical record term database in order to provide a concise list. This could be problematic, as consumer terms may not occur in physicians’ notes. Evidence for their absence is the drop in valid terms after VA medical record term filtering from 651 to 215. C-value balances termhood by not requiring prefiltering. However, for C-value to generate a concise list, too many valid terms are excluded, only 62 out of 651.

An implication of this study specific to this type of system is the choice of threshold. We found empirically that a threshold of 3.6 for the termhood score and a threshold of 15 for the C-value score produced a list that retained enough valid terms while excluding enough invalid terms. It is possible to manipulate these thresholds. Looking at Figure 1, it can be seen that a termhood threshold of 4 produces a candidate term list which is 95% valid terms. Unfortunately, the total number of valid terms found would be only 42 out of a possible 651. We consider identifying only 6% of the available terms too inefficient. Increasing the C-value threshold produces a similar result. While the valid term yield increases to 78%, only 44 valid terms are identified. It is possible that these thresholds could be increased and the number of valid terms missed could be mitigated by processing an extremely large number of webpages.

### General Implications

This system could potentially be used for vocabulary maintenance beyond CHV and even beyond the health domain. Since an increasingly large proportion of contemporary writing is published on the Internet, it is possible to crawl open-access journals, blogs, and Web news channels to identify new candidate terms for inclusion in a variety of vocabularies.

This system could also potentially be used to track the evolution of lay health language. Once the system is up-to-date, each new set of updates will be representative of the changes occurring in consumer terminology. It may be possible to use this information to recognize patients’ understanding and information needs based on their vocabulary.

### Limitations

A potential limitation of using the PatientsLikeMe website is the “higher level” language that occurs in the content produced by the site operators as opposed to the users. Higher level refers to language that is drawn directly from physicians’ vocabulary. In this case, it is likely that the term will be contained in the UMLS Metathesaurus and thus ignored in the collection of new terms. Additionally, this broader exposure to higher level language may cause increased migration of terms. The migration of such terms would be reflected in the frequency-of-use data that are used to recommend the name preferred for use in reference to the concept (the consumer preferred CHV name). Either way, this language should not present a problem for the CAU system.

The CAU system is limited by errors in the parsing and filtering stages. Although part of speech and noun phrase parsing are relatively mature NLP technologies [27], the parsing of webpages poses extra challenges due to the prevalence of incomplete and ungrammatical sentences. The parser used in the HITEx system, OpenNLP, is trained to work with general text. HITEx was developed for the processing of clinical notes, which may be more grammatical or adhere to a different subgrammar. The continuing development of the HITEx NER system incorporated into the CAU will allow it to take advantage of any advances in the parser or mapper associated with HITEx.

Filtering using the medical record data is limited by the size and clinical characteristics of the patient population represented in the medical record database. The database could be enlarged with proper institutional review board approval. The use of C-value with the unfiltered terms also decreases the effect of this limitation.

The two ATR methods [18,19] are also imperfect. Their performance could be improved by preselecting the text to the extent it is practical in the social network setting. There may be a way to target specific locations in the website or on the webpages, perhaps by searching for key section headings or HTML tags.

Another limitation of the CAU system is the continuing need for human review. The grand goal of all automated systems is to operate completely without human intervention or possibly with only minimal expert review. Our current development is far from reaching the goal of zero human review but not from the goal of minimizing reviewer time.

The results of this method on corpora other than PatientsLikeMe require further studies. To assess the potential robustness of the technique we processed the first 300 pages encountered on a crawl of the YahooHealth.com website. We found 309 potential terms with 72 terms valid for inclusion in the CHV, that is, a 23% valid term yield. This is a lower yield than from the PatientsLikeMe.com site. However, as previously discussed, the thresholds we chose impacted the yield.

### Future Research

One direction of our future efforts will be to further analyze the terms found and either map them to existing concepts or create new ones. The terms the CAU identifies are not yet included in the UMLS or CHV. It is therefore necessary to determine how to integrate them into the CHV. Since the majority of these new terms are synonyms of health concepts that already exist in the professional controlled vocabularies, it is a simple mapping to include them. However, some brand new concepts may be encountered, in which case we will utilize the characteristics of valid new consumer concepts described by Keselman et al [28] to help guide their inclusion.

In the future, we also plan to explore public participation in the collaborative review phase. In addition to discovering new terms, we plan to use live corpora to estimate the familiarity of health terms and harvest explanations.

### Conclusion

Social network data can be used to provide a living corpus, which can be mined to provide new consumer health vocabulary terms. Using ATR and dictionary lookup can narrow the candidate terms discovered to produce a concise list, which allows the vocabulary to evolve with the language without requiring a large amount of human review time.

## Multimedia Appendix 1

CAU presentation PowerPoint

## Abbreviations

ALS: amyotrophic lateral sclerosis
ATR: automatic term recognition
CAU: computer assisted update
CHV: consumer health vocabulary
HITEx: Health Information Text Extractor
IRB: institutional review board
LOINC: Logical Observation Identifiers Names and Codes
MeSH: Medical Subject Headings
ML: Machine Learning
MS: Multiple Sclerosis
N-gram: A group of n consecutive words
Non-CHV: Not contained in the Open Access Collaborative consumer health vocabulary
NER: named entity recognition
NIH: National Institutes of Health
NLP: natural language processing
OAC: open access collaborative
SNOMED: Systematized Nomenclature of Medicine
TS: terminology Systems
UMLS: Unified Medical Library System
