# Supplementary material for: Computer-Assisted Update of a Consumer Health Vocabulary Through Mining of Social Network Data
Source: J Med Internet Res. 2011 May 17;13(2):e37. doi: 10.2196/jmir.1636 (PMC3221384; doi:10.2196/jmir.1636)
Supplement: Supplementary file 1 [file jmir_v13i2e37_app1.ppt]

## Slide 1
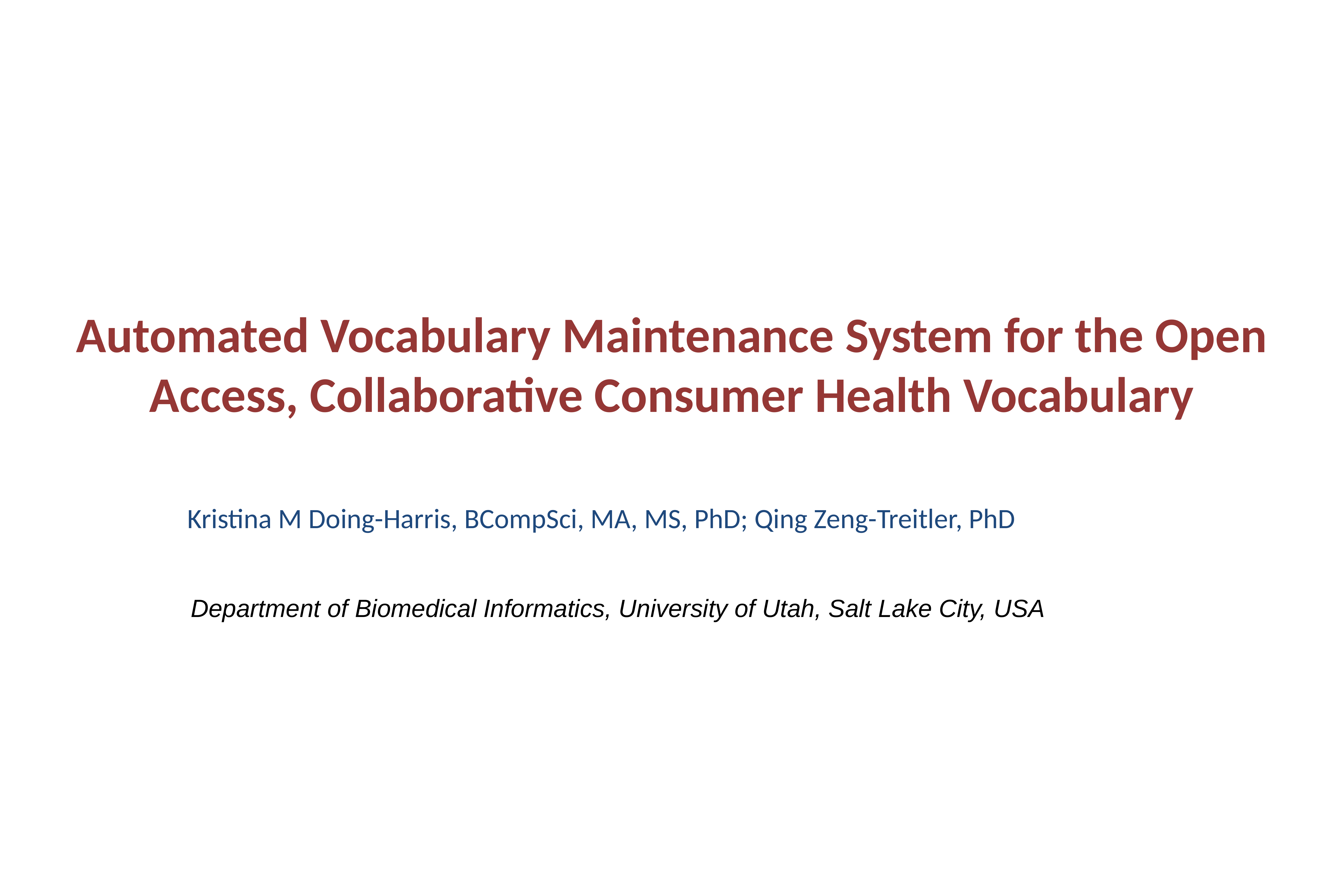

Automated Vocabulary Maintenance System for the Open Access, Collaborative Consumer Health Vocabulary
Kristina M Doing-Harris, BCompSci, MA, MS, PhD; Qing Zeng-Treitler, PhD
Department of Biomedical Informatics, University of Utah, Salt Lake City, USA

## Slide 2
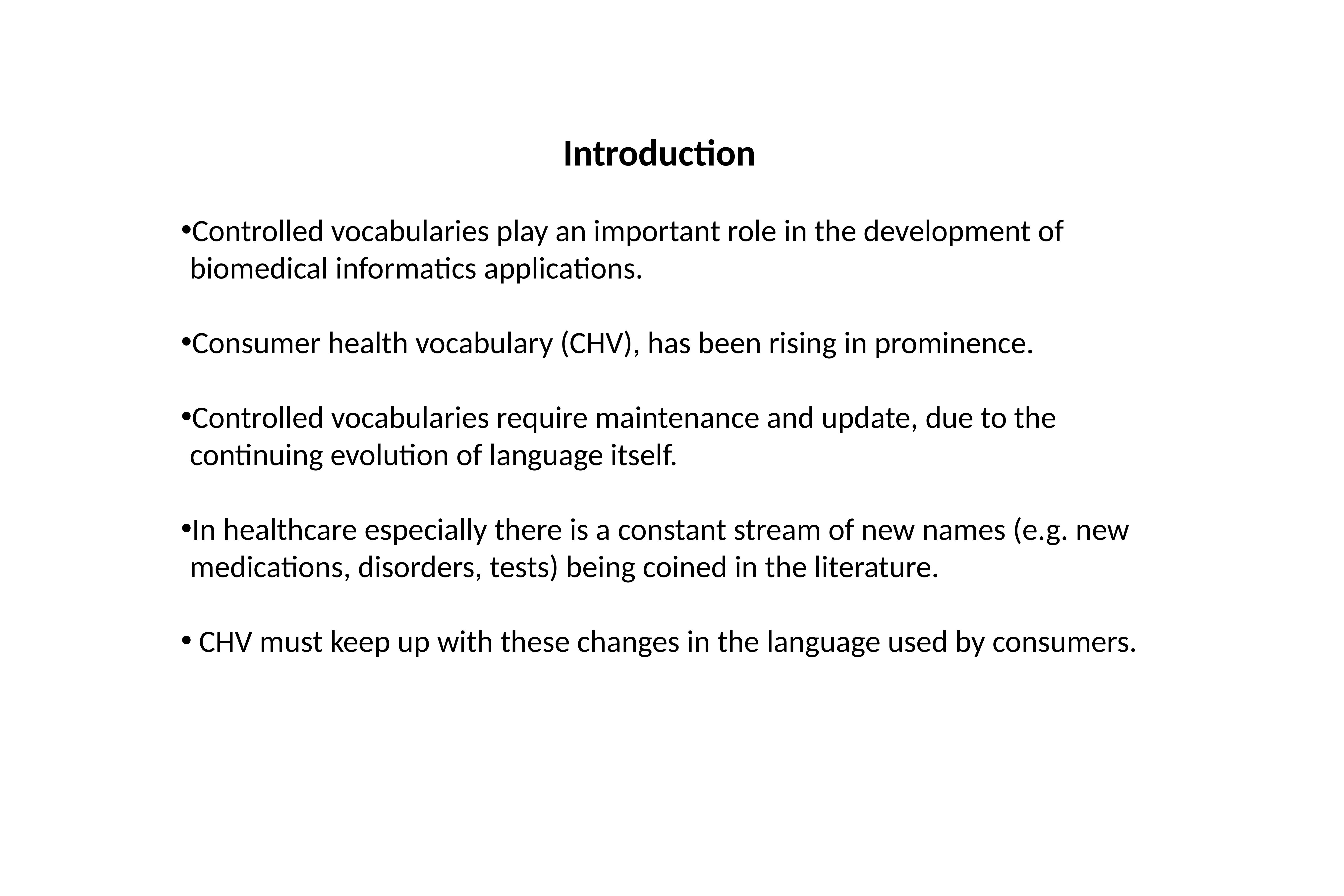

Introduction
Controlled vocabularies play an important role in the development of biomedical informatics applications.
Consumer health vocabulary (CHV), has been rising in prominence.
Controlled vocabularies require maintenance and update, due to the continuing evolution of language itself.
In healthcare especially there is a constant stream of new names (e.g. new medications, disorders, tests) being coined in the literature.
 CHV must keep up with these changes in the language used by consumers.

## Slide 3
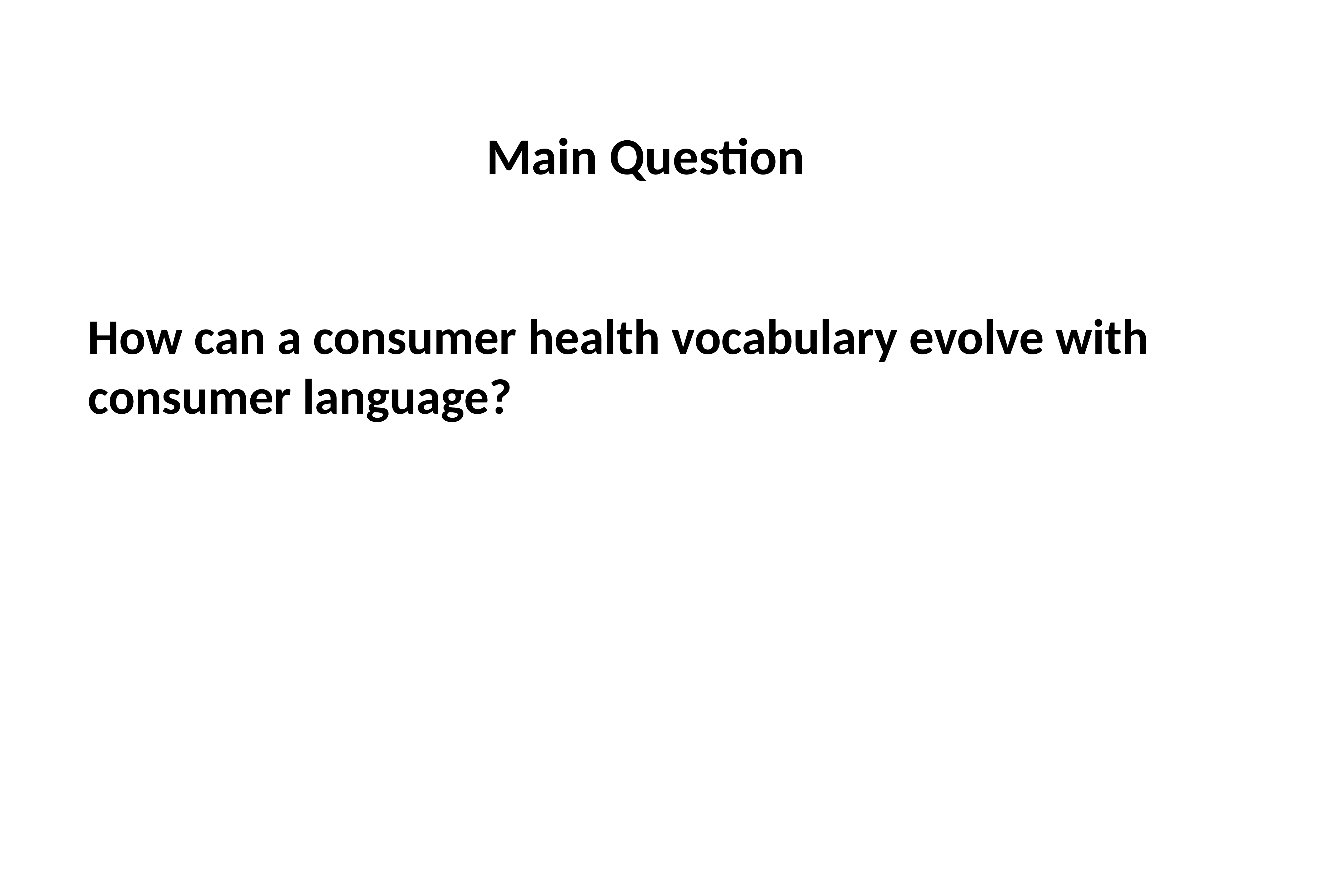

Main Question
How can a consumer health vocabulary evolve with consumer language?

## Slide 4
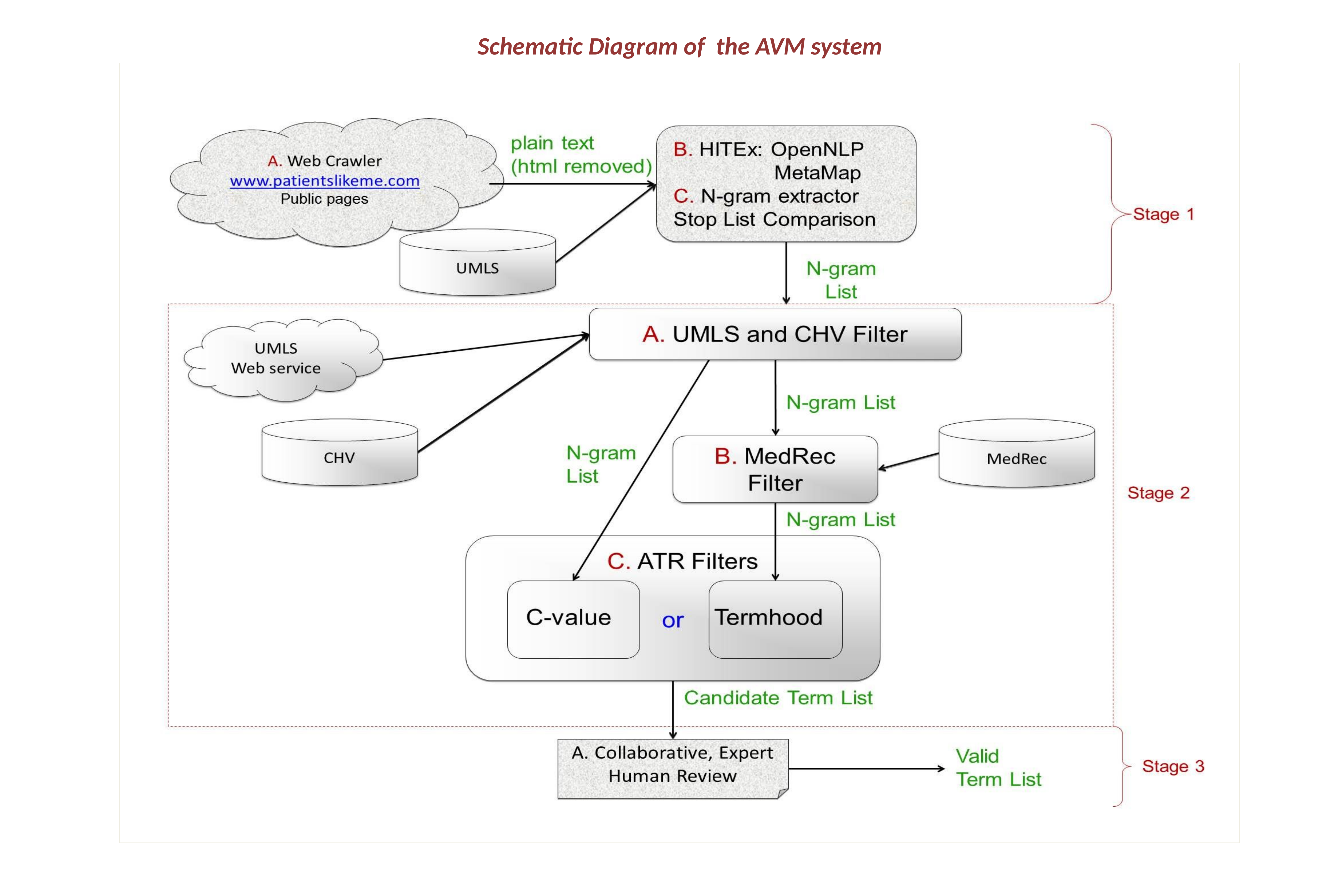

Schematic Diagram of the AVM system

## Slide 5
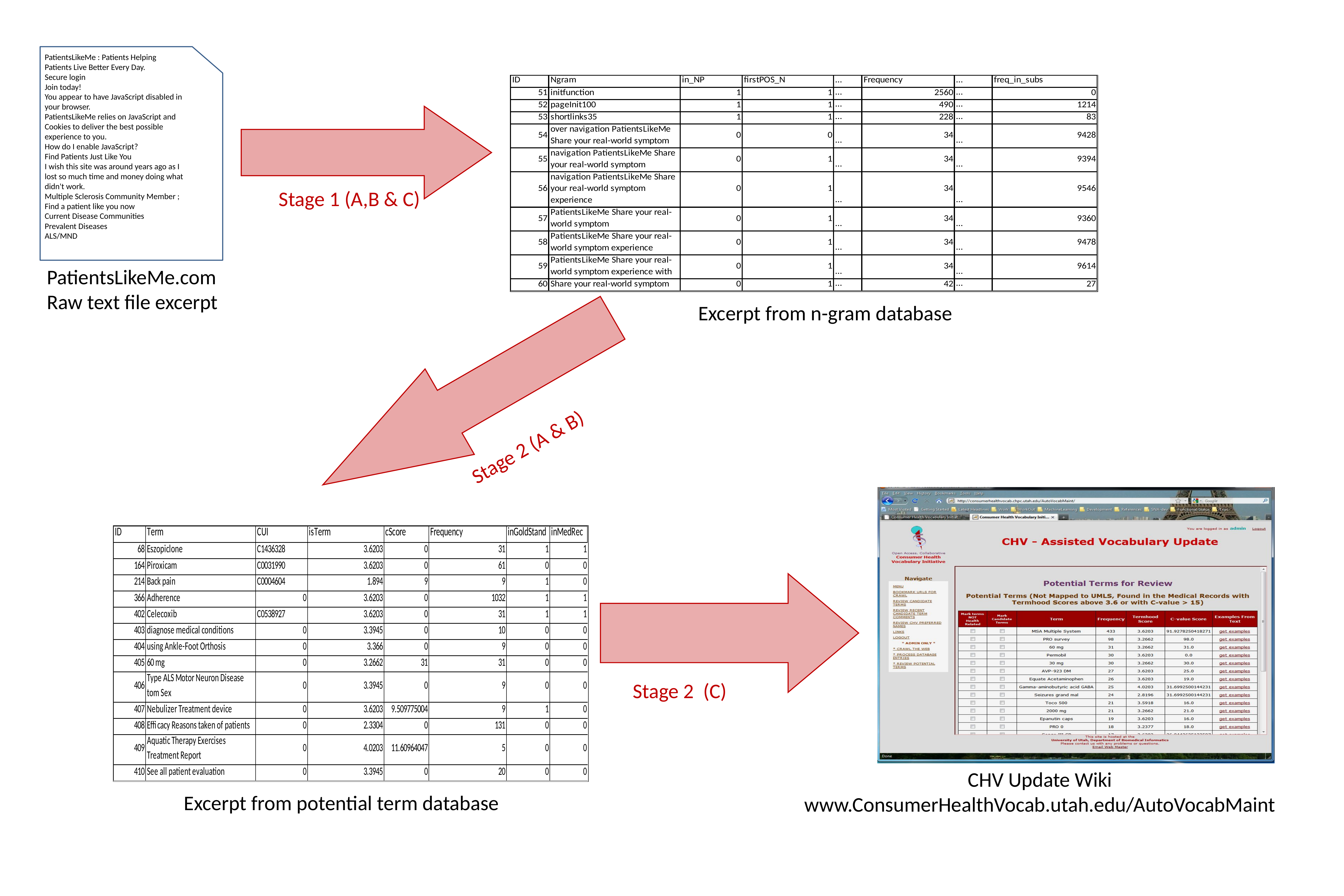

PatientsLikeMe : Patients Helping
Patients Live Better Every Day.
Secure login
Join today!
You appear to have JavaScript disabled in
your browser.
PatientsLikeMe relies on JavaScript and
Cookies to deliver the best possible
experience to you.
How do I enable JavaScript?
Find Patients Just Like You
I wish this site was around years ago as I
lost so much time and money doing what
didn't work.
Multiple Sclerosis Community Member ;
Find a patient like you now
Current Disease Communities
Prevalent Diseases
ALS/MND
PatientsLikeMe.com
Raw text file excerpt
Excerpt from n-gram database
Stage 1 (A,B & C)
Stage 2 (A & B)
CHV Update Wiki
www.ConsumerHealthVocab.utah.edu/AutoVocabMaint
Excerpt from potential term database
Stage 2 (C)

## Slide 6
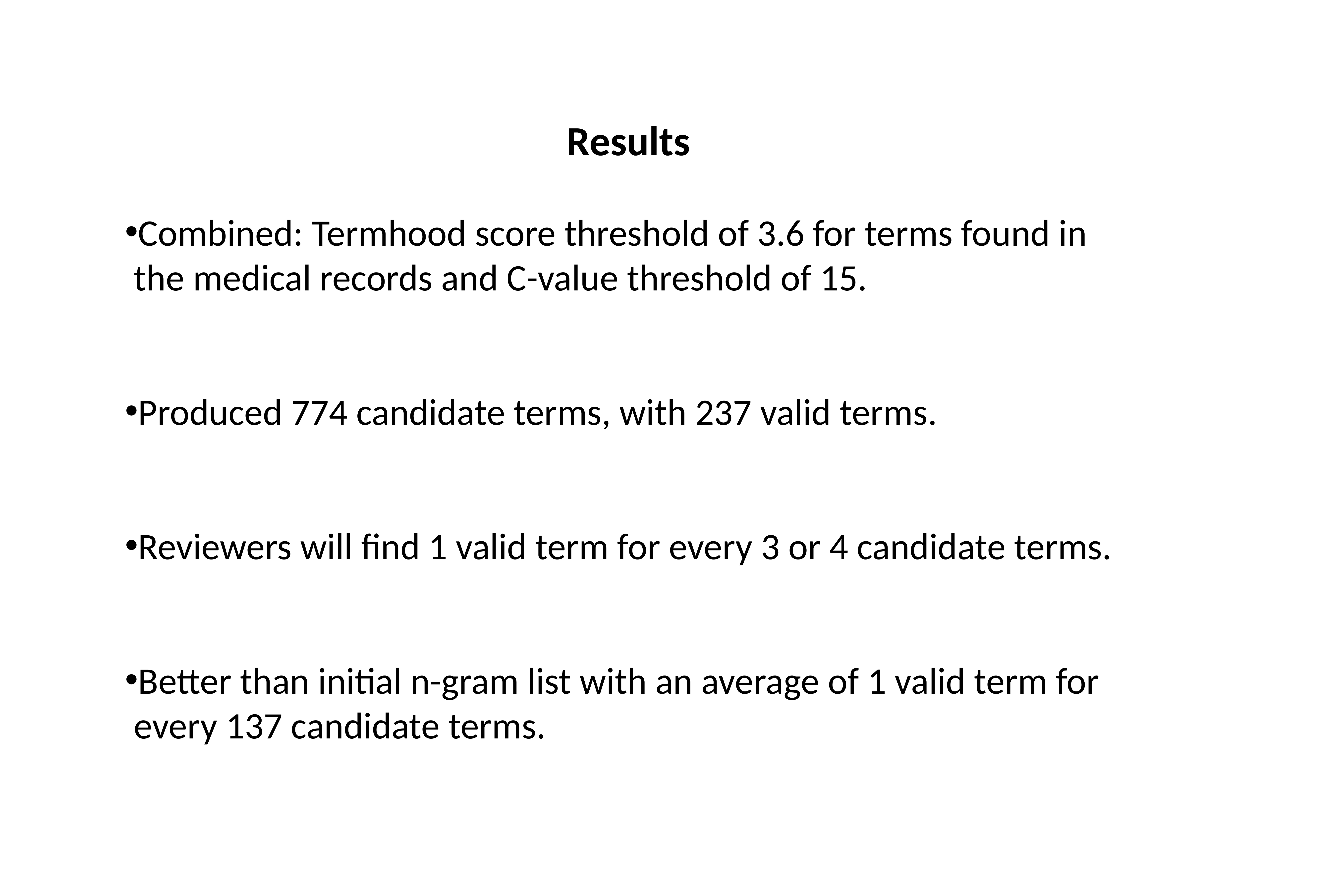

Results
Combined: Termhood score threshold of 3.6 for terms found in the medical records and C-value threshold of 15.
Produced 774 candidate terms, with 237 valid terms.
Reviewers will find 1 valid term for every 3 or 4 candidate terms.
Better than initial n-gram list with an average of 1 valid term for every 137 candidate terms.

## Slide 7
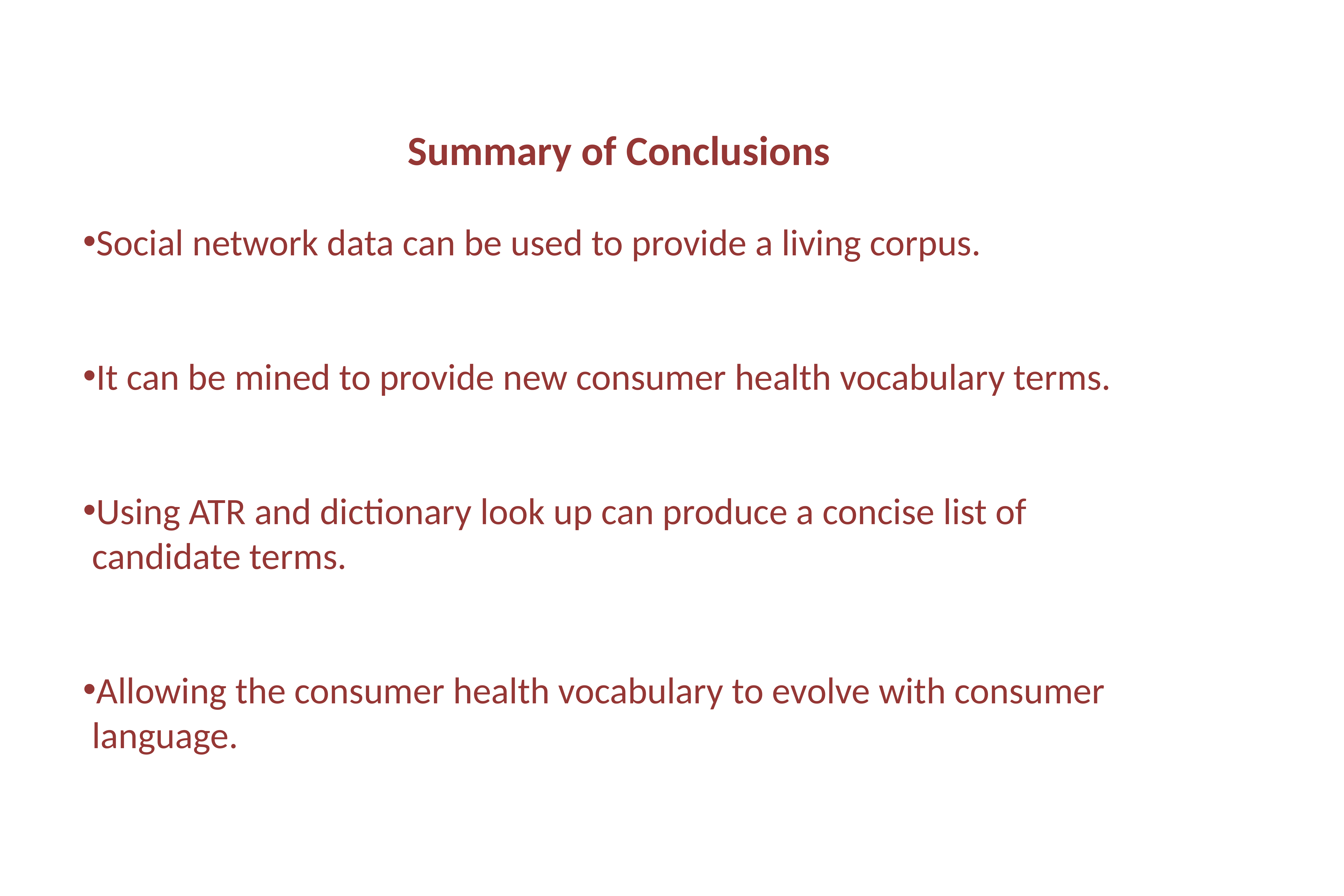

Summary of Conclusions
Social network data can be used to provide a living corpus.
It can be mined to provide new consumer health vocabulary terms.
Using ATR and dictionary look up can produce a concise list of candidate terms.
Allowing the consumer health vocabulary to evolve with consumer language.

## Slide 8
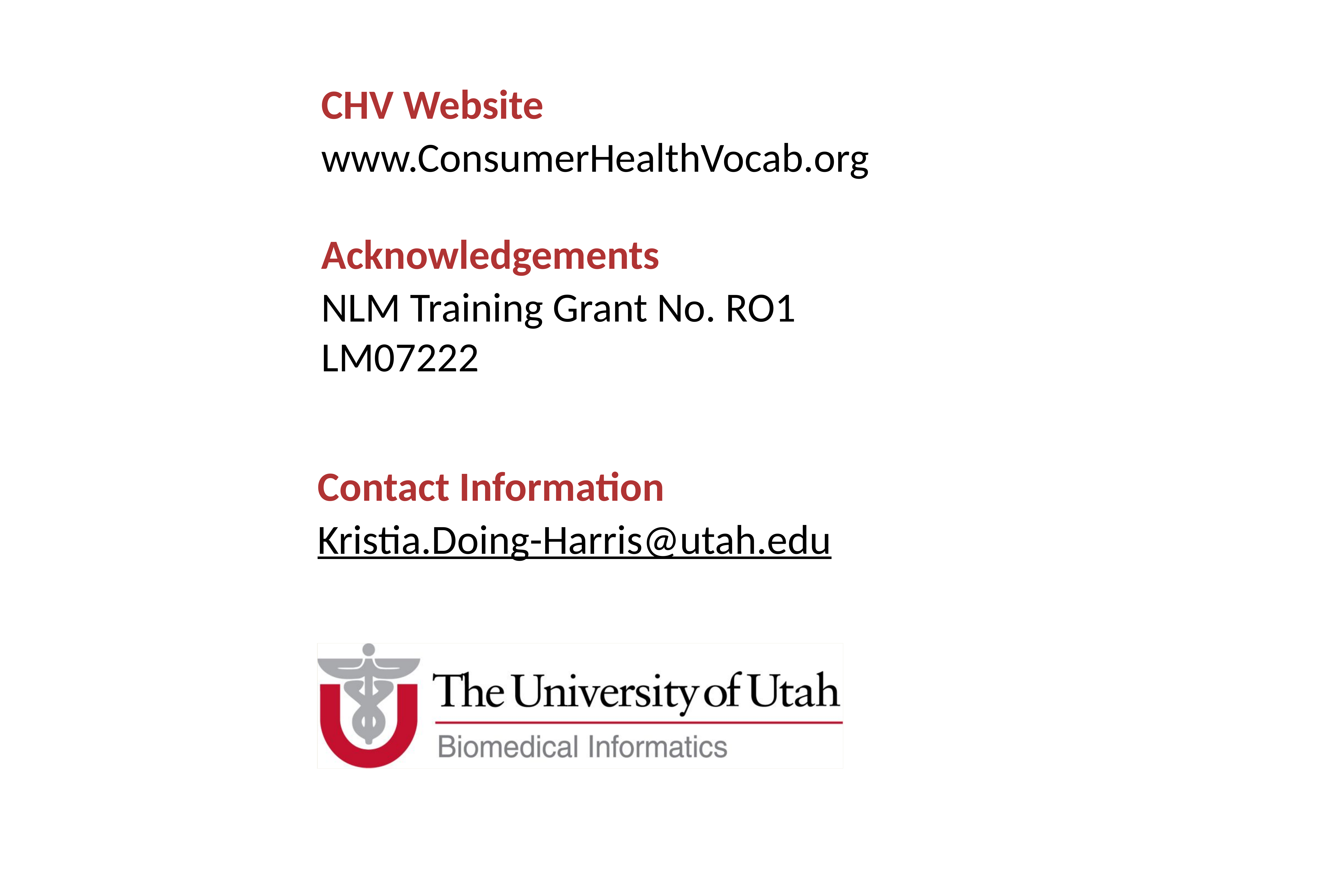

CHV Website
www.ConsumerHealthVocab.org
Acknowledgements
NLM Training Grant No. RO1 LM07222
Contact Information
Kristia.Doing-Harris@utah.edu
